# Autonomy and engagement in self-managing organizations: exploring the relations with job crafting, error orientation and person-environment fit

**DOI:** 10.3389/fpsyg.2023.1198196

**Published:** 2023-09-15

**Authors:** Maria Doblinger

**Affiliations:** Institute of Psychology, Heidelberg University, Heidelberg, Germany

**Keywords:** self-managing organization, decision autonomy, error orientation, job crafting, learning from errors, risking errors, error strain, work engagement

## Abstract

**Introduction:**

Self-managing organizations are a novel organizational form that radically decentralizes decision authority to adapt to the volatile business environment and the demands of knowledge work, resulting in new resources and demands for the employees. Therefore, building on the job demands-resources theory and the person-environment fit theory, the associations of self-managing organizations with higher perceived individual autonomy were tested. Additionally, the study investigated how job crafting and handling mistakes related to the relationship between job autonomy and work engagement/satisfaction.

**Method:**

A cross-sectional study was conducted to gather data from employees of different self-managing organizations and non-self-managing organizations, and group comparisons and path analyses were applied to test the preregistered hypotheses.

**Results:**

Increased method and decision autonomy, job crafting behaviors, error management orientation, work engagement, and job satisfaction were found in self-managing organizations. Additionally, a surplus of perceived autonomy compared to the ideal autonomy was associated with lower work engagement and job satisfaction compared to a fit between ideal and perceived autonomy. However, job crafting did not relate to a better fit between ideal and perceived autonomy. Decision autonomy predicted higher crafting of challenging demands and structural resources for employees with low error strain. Depending on the autonomy type, learning from errors enhanced or reduced the relationship between perceived autonomy and job crafting.

**Discussion:**

This study showed the importance of addressing the higher level of individual autonomy in self-managing organizations and offered starting points for interventions to support employees with handling high autonomy. Reducing error strain but increasing error learning and risking errors could help increase job crafting and work engagement, particularly in self-managing organizations.

## 1. Introduction

What if we change the game, abolish middle management, and let the employees self-manage? This question arised in the search for an adaptive strategy toward the increasingly dynamic and uncertain business environment, the employees' demand for more self-determination and purpose, and the increasing societal demand to contribute purposefully. The COVID-19 pandemic fueled all these challenges, which caused turbulences in the market but also turned upside down the daily job and family routine for many employees (Kaushik and Guleria, 2020; Shirmohammadi et al., 2022; Chung et al., 2023; Leslie-Miller et al., 2023). First studies showed that self-managing processes and flat hierarchies helped handle the COVID-19 challenges, like the sudden remote work (Maurer et al., 2022).

Self-managing organizations (SMOs) are organizations that “radically decentralize authority in a formal and systematic way throughout the organization” (Lee and Edmondson, 2017, p. 39). This organizational setup allows for faster decision-making as decisions can be made directly at the point where needed (Puranam and Håkonsson, 2015; Burton et al., 2017; Lee and Edmondson, 2017). However, engaged and healthy employees are the prerequisite for the sustainable success of any organization (Pfeffer, 2010). Initial practice reports showed that not all employees feel more engaged in SMOs (Maier, 2013; Lam, 2016; Schell and Bischof, 2022), and thus, individual-level factors seem to be important as well. As authority decentralization also implies changes in other organizational core issues, such as labor division or provision of rewards, and also in job conditions, like decision autonomy or supervisor support (Lee and Edmondson, 2017; Martela, 2019), employees in SMOs may face new job resources and demands. This may require certain changes in the employees' behaviors, attitudes, or competencies to adapt to these altered job conditions and, thus, stay engaged and healthy.

Initial research on SMOs proposed the need for specific personal characteristics, such as *proactiveness, motivation to learn*, and *accepting responsibility* (Corbett-Etchevers et al., 2019; Reitzig, 2022; Schell and Bischof, 2022). Additionally, a series of individual behavioral competencies, such as *assuming responsibility, deciding and initiating action*, or *learning from mistakes*, was identified as important (Doblinger, 2023). However, previous research did not look into the mechanism of why these personal characteristics are related to work engagement or job satisfaction in SMOs. Nonetheless, to better understand the relevance of these factors, opening the black box by looking into the interaction of employee characteristics and the job characteristics affected by the organizational changes in SMOs, e.g., person-environment fit regarding job autonomy, is necessary.

Therefore, to support employees in this novel, adaptive organizational form, a better understanding of resources and demands in SMOs is necessary, as that allows for targeted interventions to ensure the employees' long-term wellbeing. Although the decentralization of decision authority in SMOs implies changes in various job conditions, this work focuses on job autonomy in particular as it is a crucial element in the success of SMOs: on the one hand, higher autonomy, resulting from the decentralization of authority, enables those employees who are most knowledgeable to make decisions and thus get better decisions as an organization. On the other hand, this requires engaged employees, and prior research on job design showed that high levels of job autonomy were not a surefire success (Stiglbauer and Kovacs, 2018; Dettmers and Bredehöft, 2020).

Initial research on SMOs identified a range of personal traits and competencies that might be beneficial to flourish in SMOs (Corbett-Etchevers et al., 2019; Reitzig, 2022; Schell and Bischof, 2022; Doblinger, 2023). However, these studies were mainly based on qualitative methodology, impeding understanding of the effect mechanism and preventing the broader generalization. Therefore, to get insights into the operating principles of SMOs, this work investigates how proactive behaviors toward crafting one's job and the attitude toward errors relate to the associations between job autonomy and work engagement or job satisfaction in SMOs. The paper focuses on these two aspects because both characteristics were found relevant in SMOs (Schell and Bischof, 2022; Doblinger, 2023), and both are modifiable through training or cultural interventions and, thus, are of particular relevance for organizational practice.

## 2. Theory

### 2.1. Self-managing organizations and individual job autonomy

SMOs are a novel organizational form changing organizational core principles to radically decentralize authority at an organization-wide scope, thus allowing all employees to hold a specific amount of decision rights that others cannot simply overrule, which is necessary for self-management (Lee and Edmondson, 2017; Martela, 2019). These changes usually imply abolishing traditional middle management, reducing disciplinary manager-over-subordinate-power to a minimum, and shifting authority toward individual employees (Lee and Edmondson, 2017). Notably, the authority decentralization in SMOs does not mean that SMOs are hierarchy-free. However, person-related hierarchy, as known from classic organizational setups, is abolished. Instead, it is replaced by a role-based, task-related hierarchy that is not bound to specific persons and is changing over time (Laloux, 2014; Lee and Edmondson, 2017; Martela, 2019). Hence, in SMOs, individuals can still hold particular formal leadership roles, but they are bound to revocable consent from those being managed, constrained by clear boundaries, or temporarily held (Lee and Edmondson, 2017).

According to Martela (2019), SMOs also differ in several organizational core principles from traditional organizations with more centralized decision authority (hereafter non-SMOs): the responsibility of creating new tasks is shared by employees and top management, and employees allocate tasks, as they are allowed to choose the roles and tasks for which they are competent. Rewards and incentives focus on intrinsic motivating job conditions instead of monetary compensations. Frequently, a peer-based process determines payments. Employees monitor performance and accountability of each other. Conflict resolution and combating free-riding occurs among the involved employees, supported by training and methods. Furthermore, high information transparency is required to enable every employee to make decisions in the interest of the whole organization (Martela, 2019). In contrast, in non-SMOs, task identification and distribution occur in top-down processes, and broad information distribution is obsolete due to precise instructions and strict task boundaries. Moreover, supervisors allocate compensation and rewards and monitor and control work outputs (Martela, 2019). Therefore, SMOs depict a novel organizational form requiring specific consideration.

These SMO-specific changes in organizational principles also affect the team and individual levels. At the team level, for instance, decision-making processes are affected, while at the individual level, job characteristics change. For instance, authority decentralization shifts authority to the employees, and thus, the individual employee should perceive more autonomy than in traditional organizations. It is crucial to look at that, as it is the prerequisite of the intended mechanism to enable decentralized decision-making (Lee and Edmondson, 2017; Martela, 2019). Work design theory differentiates between three types of autonomy: autonomy regarding work methods, work schedules, and decisions (Morgeson and Humphrey, 2006). I expect that SMOs provide higher individual-level decision and method autonomy. The decentralization of authority allows employees to decide and choose their methods to reach a goal and thus provides employees with more decision and method autonomy than in non-SMOs. However, although this may sound obvious at first glance, it is important to empirically validate it to understand organizational behavior in SMOs, because interactions with other SMO characteristics may impede individual autonomy. For instance, Barker (1993) showed that team processes in autonomous teams restricted individual autonomy. Additionally, rigid SMO frameworks such as Holacracy, the demand for methodological synchronization, or organizational alignment could prevent employees from perceiving higher method autonomy despite the decentralization of authority (e.g., Moe et al., 2021).

Initial research on SMOs already pointed out that in SMOs, individual decision autonomy is higher than in other organizations (Doblinger and Class, 2023). However, the study did not consider method autonomy and was potentially biased by the organizational size or age of the researched organizations, as the study lacked the corresponding control variables. Thus, to understand the functioning of SMOs and their impact on employees, more information on the perceived individual autonomy in SMOs is needed. Therefore, this paper aims to test whether the desired effect of SMOs indeed occurs, independently from organizational size or tenure.

*H1: Perceived decision (a) and method (b) autonomy are significantly higher in SMOs than in non-SMOs*.

### 2.2. Job autonomy, person-environment fit, and employee wellbeing

In principle, several theories on work design and motivation attributed a positive effect to job autonomy regarding motivation, health, and wellbeing (Hackman and Oldham, 1975; Bakker and Demerouti, 2007). In particular, the well-established job demands-resources theory (JD-R) distinguishes job resources from job demands (Bakker and Demerouti, 2007). Job resources refer to those aspects that are either functional in achieving work goals, reducing the costs of job demands, or stimulating personal development, fostering motivational and buffering health-detrimental processes. In turn, job demands are those aspects of the job that require effort and come with a particular cost, and thus, strain health and energy due to effortful performance-protection strategies. The definitions of resources and demands reveal a certain flexibility: specific factors can function as resources or demands, depending on their extent and context (Bakker and Demerouti, 2017). Autonomy usually is considered a resource (Schaufeli and Taris, 2014), and there is a broad body of research confirming the positive effect of job autonomy on work engagement, job satisfaction, and wellbeing (e.g., Chung-Yan, 2010; Spiegelaere et al., 2016; Clausen et al., 2022), particularly in the case of high job complexity (Chung-Yan, 2010). Consequently, SMOs are likely to foster work engagement and job satisfaction through the provision of high job autonomy.

Nonetheless, the positive relation of job autonomy is not universal. There is also theory and research pointing to a curvilinear relationship between autonomy and work engagement or satisfaction, with the best outcomes at moderate levels of job autonomy (Kubicek et al., 2014; Stiglbauer and Kovacs, 2018). In particular, method autonomy was found to function also as job demand by increasing work intensification (Bipp et al., 2021). Additionally, the individual fit regarding ideal and perceived job autonomy is essential. Person-environment fit (P-E fit) refers to the “compatibility between an individual and a work environment that occurs when their characteristics are well matched” (Kristof-Brown et al., 2005, p. 281). The theory of P-E fit suggests that P-E fit reduces stress and turnover but increases work engagement and performance (O'Reilly III et al., 1991; Edwards et al., 1998; Kristof-Brown et al., 2005; Morrow and Brough, 2019). Several studies showed that the effect of autonomy is a question of P-E fit. Too much autonomy could have a diminishing effect on satisfaction, health, or motivation. The perception of too much autonomy depends on individual preferences and job-related expectations (Ford, 2012; Stiglbauer and Kovacs, 2018).

Ford (2012) found that job satisfaction was highest and depression lowest when the perceived autonomy matched the autonomy expected for this vocation. If it surpassed or fell below the expected level, satisfaction decreased while depression increased. That is critical for SMOs, as here, the employees hold more decision rights than in other organizations, and hence, it may exceed, in many cases, the usual autonomy in this type of vocation. Indeed, a study including employees of SMOs showed that the (mis-)fit regarding decision autonomy was related to work engagement, and in the case of fit, increases in decision autonomy were related to increases in work engagement (Doblinger and Class, 2023). Such curvilinear relations were also found between (mis-)fit and wellbeing, flourishing, and satisfaction in samples of non-SMO employees (Edwards and Rothbard, 1999; Stiglbauer and Kovacs, 2018). These findings align with the job demands-resources model that assumes that one particular aspect of a job, for instance, the autonomy level, can function as a demand or resource, depending on its level and personal resources, among others. Personal resources are aspects of the self that relate to self-efficacy and resilience (Hobfoll et al., 2003), which is the “positive adaptation, or the ability to maintain or regain mental health, despite experiencing adversity” (Herrman et al., 2011, p. 259).

In application to SMOs, this implies that the decentralization of decision authority brings the potential to increase work engagement and satisfaction [which in turn fosters sustainable performance (Pfeffer, 2010)]. As this positive effect mainly occurs when perceived autonomy fits the individual preference, identifying influencing factors for P-E fit is necessary to support employees and prevent excessive demand by their job autonomy.

### 2.3. Job-crafting as behavior to cope and deal with job autonomy

Proactive behavior was relevant in SMOs and other work contexts with high authority decentralization (Dettmers and Bredehöft, 2020; Doblinger, 2021, 2023; Schell and Bischof, 2022). Thus, it may also be a helpful behavior to achieve a better person-environment fit regarding job autonomy in SMOs. One well-established type of proactive behavior in the context of job design research is *job-crafting*, a bottom-up process of job design, referring to the proactive modification of one's work (Wrzesniewski and Dutton, 2001; Tims et al., 2012). Job crafting was found to be related to increased work engagement, satisfaction, performance, wellbeing (Bakker et al., 2012; Tims et al., 2013; Vogt et al., 2016), and person-environment fit (Chen et al., 2014; Tims et al., 2016; Kooij et al., 2017; Wang et al., 2017). Scholars focused on different job-crafting subjects, for instance, different types of resources or job-related cognitions (Wrzesniewski and Dutton, 2001; Tims et al., 2012; Zhang and Parker, 2019). The current work considers Zhang and Parker's concept of behavioral approach crafting, which refers to actions to increase one's job resources and challenging demands, which are particularly relevant in SMOs because it includes elements of self-management and bottom-up task creation (Martela, 2019).

Different relations between work engagement and job crafting have been proposed (Demerouti, 2014). One stream of research focused on the positive effect of job crafting on work engagement (Bakker et al., 2012; Petrou et al., 2012; Vogel et al., 2016; van Wingerden et al., 2017). For instance, according to Moreira et al.'s (2022) study, job crafting behaviors were positively related to work engagement mediating the relationship between crafting social resources and challenging demands with job performance. However, this effect was not found for structural resources. In contrast, another stream focused on the positive impact of work engagement on job crafting, arguing that job crafting is how engaged employees create resources increasing engagement over time and creating gain spirals (Bakker, 2011). Looking at SMOs, where proactive behaviors have been identified as important, this work focuses on the effect of job crafting on work engagement. Job crafting allows for modifying job resources and demands and thus can increase the person-environment fit (Tims et al., 2016; Kooij et al., 2017; Wang et al., 2017). Misfit was also considered as a trigger for job crafting, potentially in order to increase fit. For instance, Dust and Tims (2020) showed increased job crafting behaviors when the interdependence supply exceeded or failed the individual interdependence need. Additionally, the effect was increased by autonomy. Therefore, I argue that job-crafting behaviors predict work engagement and job satisfaction in SMOs because they help increase the person-environment fit regarding job autonomy and, thus, enhance the positive effect of job autonomy on work engagement and satisfaction.

Additionally, I suggest job crafting in terms of behavioral approach crafting is more important and prevalent in SMOs than in traditional organizations. As the decision and method autonomy is unusually high, potentially resulting in autonomy excess, the P-E-fit-increasing role of job crafting is more important. Secondly, higher job autonomy was associated with more job-crafting behavior (Petrou et al., 2012; Rudolph et al., 2017; Kim et al., 2018). Additionally, previous research showed that job crafting was used to cope with new situations and handle organizational change (Kira et al., 2010, 2012) and positively related to readiness for change (Lyons, 2008). Hence, this work is aimed at testing the following hypotheses:

*H2: Job crafting predicts higher job satisfaction and work engagement, mediated by better P-E fit regarding decision autonomy (a) and method autonomy (b)*.

*H3: Job crafting is more critical in SMOs than in other organizations*.

### 2.4. Error orientation

Prior research showed that *handling mistakes constructively* was an important individual competency in SMOs (Doblinger, 2023) and may also interact with individual job autonomy. Constructively handling mistakes means that mistakes are considered an opportunity to learn instead of mere failure. Thus, the risk of mistakes is also accepted to increase knowledge and advance in uncertain conditions. Although prior research investigated the orientation toward mistakes often at the organizational level (Dahlin et al., 2018), Rybowiak et al. (1999) developed the error orientation questionnaire to measure how individuals perceive and appraise mistakes. The questionnaire is based on eight dimensions, including risking errors, learning from errors, strain from errors, thinking about errors, covering up errors, or error anticipation^1^. The dimensions of risking errors, learning from errors, and strain from errors reflect critical aspects of handling mistakes constructively, and thus their role in SMOs regarding handling job autonomy might be of particular interest. Previous literature used *error management orientation* or simply *error orientation* when referring to constructively handling mistakes (Rybowiak et al., 1999; van Dyck et al., 2005; Keith and Frese, 2008). Therefore, in the following, *error orientation* refers to the constructive handling of mistakes.

Research on the relation of individual error orientation with work engagement or job autonomy is still lacking. However, individual-level error orientation related positively to opportunity identification, entrepreneurial decision-making, and performance under uncertainty (Arenas et al., 2006; Wei and Hisrich, 2016; Roose, 2018), which are both important behaviors in SMOs (Martela, 2019; Doblinger, 2023), and likely, for handling high decision autonomy. Additionally, the comparison of managers and employees regarding error orientation showed a stronger appraisal of mistakes as learning opportunities by managers than employees; however, there was no difference regarding mistake-related strategies or emotions (Harteis et al., 2008). Additionally, Loh et al. (2013) showed an effect of training interventions by finding increased performance after error management training, while error avoidance training decreased performance. Moreover, team and organizational-level error orientation was positively related to performance and innovation (van Dyck et al., 2005; Tjosvold and Yu, 2007; Putz et al., 2013; Javed et al., 2020).

Job autonomy in SMOs requires the individual to make decisions—even under uncomfortable uncertainty, but the delegation to one's leader, as done in more hierarchical organizations, is not possible or accepted anymore. Hence, to make decisions under uncertainty, accepting the risk of making errors is necessary (Tjosvold and Yu, 2007). Due to the natural tendency toward risk aversion, this is a challenging point in SMOs.

Having a positive attitude toward mistakes can become a personal resource, as it links to resilience (Hobfoll et al., 2003; Xanthopoulou et al., 2009). I argue that error management orientation in terms of learning from errors, risking errors, and low strain from errors is relevant to the effect of job autonomy on work engagement. Error orientation functions as a personal resource in the context of SMOs as it can strengthen personal resilience (Xanthopoulou et al., 2009; Herrman et al., 2011). The decision authority employees receive in SMOs leads to high job autonomy and consequently requires the corresponding decisions. Making decisions seems more manageable when the fear of making mistakes is low, and mistakes are considered learning opportunities (Wei and Hisrich, 2016; Metcalfe, 2017). Therefore, I expect that individuals with a positive attitude toward mistakes will benefit more from high levels of autonomy than individuals struggling with making mistakes, and thus, I propose the following hypothesis:

*H4: Error orientation in terms of taking error risks (a), learning from errors (b), and low error strain (c) increases decision and method autonomy's positive relationship with work engagement and job satisfaction*.

In addition, I also expected an enhancing effect of error orientation on job crafting. Prior research provided evidence of a positive relationship between learning from mistakes or error risk acceptance and job crafting. For instance, error orientation was positively associated with readiness for change and personal initiative (Rybowiak et al., 1999), and job crafting can be considered a specific type of personal initiative. Besides, individual error orientation was found to be positively related to job crafting, mediated through personal growth initiative (Fischer, 2021). According to the regulatory focus theory (Higgins, 1997), humans seek pleasure but avoid pain through behaviors to either avoid pain or promote pleasure. Linking that to error orientation, the dimensions of error orientation represent either an avoidance focus, which includes concentrating on safety, responsibilities, and avoiding losses (e.g., error strain, cover-up), or a promotion focus, which includes hopes, accomplishments, and gains while pursuing their goals (e.g., learning from errors, error risk). Research on regulatory focus showed that a general prevention focus predicted more hindrance demands reduction and prevention-focused job-crafting, while a promotion focus predicted promotion-focused job crafting, including increasing challenging demands and resources (Rudolph et al., 2017; Lichtenthaler and Fischbach, 2019). Similarly, approach temperament was positively related to seeking resources and demands, while avoidance temperament was positively related to reducing demands (Bipp and Demerouti, 2015). Hence, based on the assumption that learning from mistakes and risking errors align with a promotion focus while strain from errors aligns with a prevention focus, I assume that they predict job crafting behavior to increase resources and challenging demands. Higher job autonomy also predicted more job crafting behavior (Petrou et al., 2012), so I expect an interaction between job autonomy and error management orientation. Consequently, the following hypotheses are proposed:

*H5: Learning from errors (a), taking error risks (b), and low error strain (c) enhance the positive relation between decision (method) autonomy and job crafting*.

In SMOs, individual job autonomy and the requirement for proactive behaviors are higher, so handling uncertainties and making decisions that could turn out to be wrong is more often necessary. Having an error management orientation is helpful in this case because it reduces the burden of potentially harmful decisions. Additionally, previous literature showed the importance of constructively handling mistakes in SMOs (Doblinger, 2023). I also expect risking errors, learning from errors, and low error strain to be more important in SMOs than in other organizations.

*H6: Error orientation in terms of learning from errors (a), taking error risks (b), and low error strain (c) is more critical in SMOs than in other organizations*.

## 3. Method

All hypotheses have been pre-registered before complete data collection (https://doi.org/10.17605/OSF.IO/9SQWU).

### 3.1. Sample

In order to include employees from SMOs and non-SMOs, the participants were recruited through two different approaches. Firstly, employees of SMOs were recruited through direct contact with SMOs. Secondly, employees of non-SMOs were recruited through social media platforms and the research panel Prolific (Prolific, 2022). The final sample consisted of 278 participants in total. Although the planned sample size (non-SMOs and SMOs) was at least 100 participants of each group, due to participant burden and losses in the data cleaning phase, the study relied on a smaller sample of SMO employees (*n*_*SMO*_ = 78, *n*_*non*−*SMO*_ = 167, *n*_*other*_ = 33). However, the subsample was large enough to make the planned group comparisons. The characteristics of the sample are displayed in detail in Table 1. All participants had the option to receive customized feedback on their answers regarding job crafting and error orientation, and participants of the research panel additionally received financial compensation of 0.75£ (= 9.00£/h; amount suggested by the panel provider).

**Table 1:** Sociodemographic characteristics of the sample.

	**All**	**SMO**	**non-SMO**
**Age**			
19 years or younger	2	1	1
20–24 years	12	3	6
25–29 years	52	12	33
30–34 years	51	12	33
35–39 years	41	12	22
40–44 years	29	10	17
45–49 years	22	8	12
50–54 years	25	9	14
55–59 years	24	7	13
60–64 years	9	1	8
65 years or older	6	1	5
**Gender**			
Female	155	42	89
Male	111	34	68
**Leadership**			
No leadership responsibility	132	33	86
Leadership responsibility	145	44	81
**Org size**			
Microenterprise	19	8	10
Small and medium-sized enterprises	95	27	55
Large enterprise	135	40	77
**Organizational age**			
<1 year	21	2	18
1–2 years	9	38	7
2–5 years	61	37	15
>5 years	186		127
**Business sectors**			
Administration	11	0	11
Automotive	21	4	17
Architecture/construction	10	1	9
Consulting	9	6	3
Education	19	1	18
Chemistry	2	0	2
Services	5	2	3
IT	47	37	10
Finance	9	0	9
Research and development	9	0	9
Retail	10	5	5
Industry/manufacturing	9	0	9
Health	30	11	19
Food/agriculture	1	1	0
Public administration	3	0	3
Human resources	1	0	1
Legal	2	0	2
Others	10	1	9
Social institutions	23	9	14
Tourism/hospitality	3	0	3
Logistics/transport	4	0	4

Numbers depict the absolute amount of persons with this value.

### 3.2. Procedure

Participants were invited to answer the online questionnaire via direct contact with SMOs, social media platforms, and the prolific research platform. In order to incentivize participation, automated personalized feedback on one's answers to the questionnaire was offered. After confirming the informed consent, participants answered the items on perceived and ideal autonomy, job crafting, error orientation, work engagement, job satisfaction, self-managing, and more general organizational characteristics of the current employer. Participants were also asked about their work experience in SMOs and, in general, their job position, potential leadership role, and working hours in an employment relationship. The leadership role was assessed by asking the participants whether they held a specific leadership responsibility, such as processual or technical. In the end, participants were asked to provide information on their sociodemographic characteristics. After finishing the questionnaire, the participants could receive feedback on their answers regarding job crafting and error orientation, and the feedback was displayed accordingly.

Common source bias was encountered by several procedural remedies, recommended by Podsakoff et al. (2003): (1) participants were informed that answers were anonymous and desired to be as honest as possible as there was no correct answer; (2) question order was counterbalanced by alternating questions measuring the predictor and questions measuring the criterion; (3) variations in response scales; (4) the items in use were checked to fulfill the criteria of clarity, unambiguity and simplicity; and (5) mid- and endpoints of scales were labeled.

### 3.3. Measures

#### 3.3.1. Ideal and perceived decision and method autonomy

Ideal and perceived decision and method autonomy were assessed by the corresponding six items of the German and English versions of the Work Design Questionnaire (Morgeson and Humphrey, 2006; Stegmann et al., 2010). The items (e.g., “The job allows me to make a lot of decisions on my own”) were rated on a 5-point scale ranging from *not at all* to *completely*. Following Stiglbauer and Kovacs's (2018) approach, the items to measure ideal and perceived decision-making autonomy were the same but introduced by two different questions: the items on perceived autonomy were introduced by the question “To what extent does this apply to your current job?” whereas the items on ideal autonomy were introduced by the question “To what extent does this apply to your ideal job?”.

#### 3.3.2. Error orientation

Taking error risks, learning from errors, and low error strain were measured by using the corresponding scales of Rybowiak et al. (1999). A sample item of the four items measuring taking error risks was “If one wants to achieve at work, one has to risk making mistakes”. A sample item of the four items to measure learning from errors was “Mistakes assist me to improve my work”. A sample item of the four items to assess error strain was “I am often afraid of making mistakes”. All items were rated on a 5-point Likert scale ranging from *not at all* to *completely*.

#### 3.3.3. Job crafting

In order to assess the job-crafting dimensions of increasing structural job resources, increasing challenging job demands, and increasing social job resources, the corresponding scales of Lichtenthaler and Fischbach (2016) (based on Tims et al., 2012) were used. All items were rated on a 5-point Likert scale, ranging from *never* too *often*. Five items measured increasing structural job resources, and a sample item was “I try to learn new things at work”. Five items measured increasing challenging job demands, and a sample item was “When an interesting project comes along, I offer myself proactively as a project coworker”. Five items measured increasing social job resources, and a sample item was “I ask others for feedback on my job performance”. The items of this scale were adapted to the framework of self-management by extending the term “supervisor” to “supervisor/colleagues”.

#### 3.3.4. Work engagement

For assessing work engagement, the three-item ultrashort Utrecht Work Engagement Scale (UWES-3) to measure work engagement was used (Schaufeli et al., 2019). Three items measured each dimension of work engagement, vigor, dedication, and absorption. Each item was rated on a seven-point scale ranging from *never* (1) to *always* (7).

#### 3.3.5. Job satisfaction

Job satisfaction was assessed by two items based on the scale of Kovacs et al. (2018). Initially, it was presented as a reliable 1-item scale, but in order to prevent biased results through input errors, the item was repeated in its negative version at another position in the questionnaire. “I'm satisfied with my job” was a sample item, and both items were rated on a 7-point Likert scale.

#### 3.3.6. Self-managing organization

In order to assess whether the participants worked in an SMO, the checklist approach of Doblinger and Class (2023) was taken. Participants answered a checklist with seven statements on the organization based on the characteristics of SMOs by Martela (2019). Every statement that applied to the participant's current employer should be confirmed by ticking it. Subsequently, participants additionally evaluated whether they worked in an SMO. Participants were informed that all the criteria mentioned above must be met in the case of an SMO. The checklist contained the following items: (1) the organizational hierarchy is flat; (2) decisions are not always made centrally by managers, but instead employees can make decisions on their own responsibility (decentralized decision-making); (3) not only managers but also employees define and create new tasks; (4) employees themselves decide which tasks they will work on; (5) performance control occurs mainly mutually among employees; (6) there are explicit conflict resolution mechanisms that do not require a disciplinary manager; (7) there is a high level of information transparency to enable employees to make decisions. Only those cases for which the subsequent confirmation of SMO was in line with the checklist were counted as a case of the SMO subsample in this study.

### 3.4. Analysis

**Figure 1:**
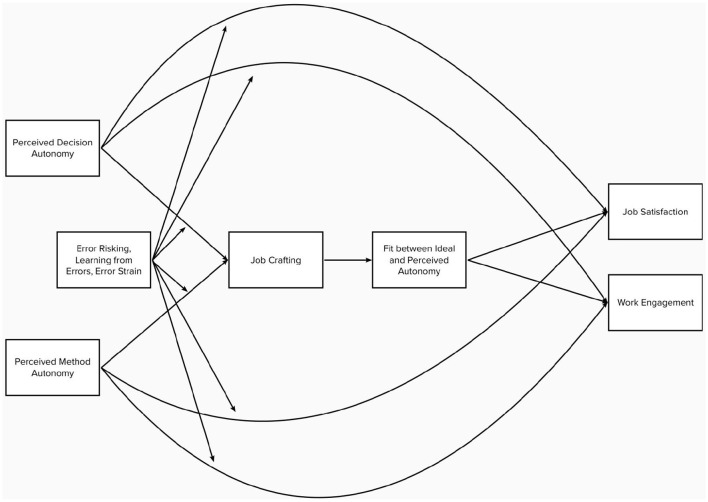
Conceptual model.

All analyses were done using the statistical software R (R Core Team, 2022). The group comparison required to test H1, was done by analyses of covariance (ANCOVA). The conceptual model (as presented in Figure 1) was divided into different parts to limit model complexity. First, the impact of job crafting on work engagement and job satisfaction, mediated through autonomy fit, was analyzed in a separate path model, and in a second path model, the moderating role of error orientation on job crafting and work engagement/job satisfaction was investigated. Given the sample size, I relied on path analysis with manifest variables because latent interactions require a larger sample size. All path models were estimated using a covariance-based approach using maximum likelihood with robust standard errors as estimation method. Four path models with ideal and perceived decision (method) autonomy, their polynomials, and the interaction term of both variables as mediators of the relationship between job crafting behaviors and work engagement (job satisfaction) were estimated to test H2. H3 and H6 were tested using group comparisons. H4 and H5 were tested based on an additional moderated mediation path model, including the interactions of error orientation dimensions and decision/method autonomy as predictors of job crafting and work engagement/job satisfaction. For the group comparisons car package (Fox and Weisberg, 2019) and for the path models the lavaan package (Rosseel, 2012) were used. The response surfaces were analyzed using the RSA package (Schönbrodt and Humberg, 2023).

The data were screened for cases signaling insufficient attention by evaluating the attention checks, the control question (“Can we use your data?”), and a relative speed index (Leiner, 2019). Only those cases in which the last survey page was reached were considered. In line with the suggestion of the research panel Prolific, one failed attention check was accepted as long as the relative speed index was not significantly increased (as suggested by Leiner, 2019), resulting in two deletions and a final sample of *N* = 278. Additionally, we examined the answers regarding SMO. The data showed some inconsistencies regarding the answers to whether the organization was an SMO or not. Some participants did choose none or only a few characteristics of SMOs but confirmed that their organization was an SMO. Different interpretations were possible but also speculative. In order to ensure good data quality for the group comparisons, the ambiguous cases were coded as a third, additional group, which was the case for *n* = 25. Additionally, eight cases in which the participants said they could not judge were also assigned to the third group, resulting in *n*_other_ = 33.

## 4. Results

Table 2 shows the correlations of all variables. All reliability scores were acceptable (Cronbach's alpha > 0.70). 12-factor confirmatory factor analysis using the lavaan package (Rosseel, 2012) showed that the measurement model with the distinct but related variables of ideal and perceived decision and method autonomy, crafting structural and social resources, crafting challenging demands, risking errors, learning, and strain from errors, work engagement, and job satisfaction fitted well with the data (CFI = 0.92; RMSEA = 0.049). Item loadings were also all in the expected direction and significant at the *p* < 0.001 level.

**Table 2:** Means, standard deviations, correlations, and reliabilities between the study variables.

		***M***	***SD***	**1**	**2**	**3**	**4**	**5**	**6**	**7**	**8**	**9**	**10**	**11**	**12**	**13**	**14**
1	Ideal decision autonomy^a^	4.26	0.81	(0.89)													
2	Perceived decision autonomy	3.86	0.92	0.60^***^	(0.88)												
3	Ideal method autonomy^a^	4.36	0.80	0.87^***^	0.54^***^	(0.90)											
4	Perceived method autonomy	3.96	0.93	0.56^***^	0.82^***^	0.59^***^	(0.87)										
5	Work engagement^a^	5.16	1.07	0.35^***^	0.39^***^	0.38^***^	0.40^***^	(0.85)									
6	Job satisfaction	5.25	1.31	0.26^***^	0.43^***^	0.31^***^	0.45^***^	0.71^***^	(0.95)								
7	Error learning^a^	3.94	0.72	0.29^***^	0.24^***^	0.32^***^	0.23^***^	0.23^***^	0.21^***^	(0.85)							
8	Error risking^a^	3.61	0.84	0.31^***^	0.23^***^	0.33^***^	0.27^***^	0.28^***^	0.24^***^	0.53^***^	(0.80)						
9	Error strain^a^	2.89	0.84	−0.18^***^	−0.2 ^***^	−0.15^*^	−0.20^***^	−0.24^***^	−0.18^***^	−0.17^*^	−0.24^***^	(0.81)					
10	Structural resources crafting	4.03	0.63	0.50^***^	0.47^***^	0.53^***^	0.46^***^	0.46^***^	0.39^***^	0.41^***^	0.44^***^	−0.21^***^	(0.76)				
11	Challenging demands crafting^a^	3.49	0.83	0.40^***^	0.29^***^	0.40^***^	0.32^***^	0.43^***^	0.26^***^	0.30^***^	0.42^***^	−0.17^***^	0.62^***^	(0.80)			
12	Social resources crafting	3.30	0.85	0.14^*^	0.12.	0.17^*^	0.18^***^	0.19^***^	0.16^*^	0.19^***^	0.08	0.15^*^	0.34^***^	0.33^***^	(0.81)		
13	Age	–	–	0.02	−0.05	0.00	−0.07	0.05	−0.02	−0.02	0.07	−0.16^*^	−0.03	0.03	−0.25^*^	–	
14	Male gender	–	–	−0.04	−0.01	−0.09	−0.05	−0.12	−0.07	0.00	0.03	−0.20^***^	−0.15^*^	−0.11	−0.28^*^	0.15^*^	–
15	Leadership responsibility	–	–	0.14^*^	0.13^*^	0.11	0.08	0.18^*^	0.01	0.03	0.21^*^	−0.15^*^	0.16^*^	0.23^***^	0.02	0.16^*^	0.05

Reliabilities in ().

^a^reduced N = 277, otherwise N = 278. ^*^p < 0.05; ^***^p < 0.001.

In addition to the procedural remedies regarding common source bias, *post hoc* statistical control was taken. Common source bias was tested by the unmeasured latent factor technique recommended by Podsakoff et al. (2012). The comparison of the standardized regression weights of the model with a common latent factor and the model without one did not point to a common method bias for most variables, except for perceived decision and method autonomy, for which the differences between the indicators exceeded the level of 0.20. Common method bias can inflate bivariate correlations and, thus, reduce the reliability of the results. However, interaction and quadratic effects can only be deflated in case of severe common source bias (Siemsen et al., 2010). In the current study, mainly interactions with perceived autonomy were tested. Thus, the common method bias is less problematic. The impact of common method bias on H1 should also be neglectable as the bias should have affected both groups (SMOs and non-SMOs) similarly. Therefore, in the current study, common source bias could not increase the type I error rate, only the type II error rate, which was tolerated acknowledging that *post hoc* statistical control also comes with several disadvantages (Conway and Lance, 2010). Analyses of statistical power showed that the power was for all relevant analyses at an acceptable level of 1 – ß > 0.80.

### 4.1. Hypothesis 1

In order to test H1a (higher perceived decision autonomy in SMOs), the group means were compared using the ANCOVA method. Gender, age, leadership responsibilities, organizational age, and organizational size were used as covariates. The results showed that perceived decision autonomy was significantly and moderately higher in SMOs (*n* = 78, *M* = 4.20, *SD* = 0.77) than in non-SMOs (*n* = 167, *M* = 3.68, *SD* = 0.96), *F*_(1, 206)_ = 13.10, *p* < 0.001, Cohen's *d* = 0.58. Therefore, H1a was confirmed. In order to test H1b (higher perceived method autonomy in SMOs), the same statistical approach and covariates were applied. Perceived method autonomy was significantly and moderately higher in SMOs (*n* = 78, *M* = 4.28, *SD* = 0.76) than in non-SMOs (*n* = 167, *M* = 3.77, *SD* = 0.98), *F*_(1, 206)_ = 11.26, *p* < 0.001, Cohen's *d* = 0.56. Therefore, H1b was confirmed.

### 4.2. Explorative analysis regarding fit

The analysis of the shares of autonomy excess, autonomy shortage, and autonomy fit showed that SMOs had more cases of congruence between ideal and perceived decision and method autonomy but fewer cases of autonomy shortage (Table 3). In turn, the cases of autonomy surplus were even a little higher in non-SMOs, where the total level of autonomy was lower.

**Table 3:** Autonomy surplus, fit and shortage in the sample and subsamples.

	**Decision autonomy**			**Method autonomy**		
	all	SMOs	non-SMOs	all	SMOs	non-SMOs
Surplus	21%	21%	23%	19%	18%	20%
Congruence	56%	65%	49%	57%	67%	51%
Shortage	23%	14%	28%	23%	15%	29%

The cutpoints for congruence were |Δz| <0.5.

### 4.3. Hypothesis 2

In order to test H2a and b, predicting job satisfaction and work engagement through job crafting, mediated by better decision (method) autonomy fit, path analysis was used. Following Edward's recommendations for analyzing fit, perceived and ideal decision (method) autonomy, their squared terms and the terms of their interactions were used as mediators, and the job crafting dimensions as predictors. Due to the small sample size, separate models were run for method and decision autonomy, work engagement, and job satisfaction. For the resulting four models, fit indices were good (CFI > 0.97).

**Figure 2:**
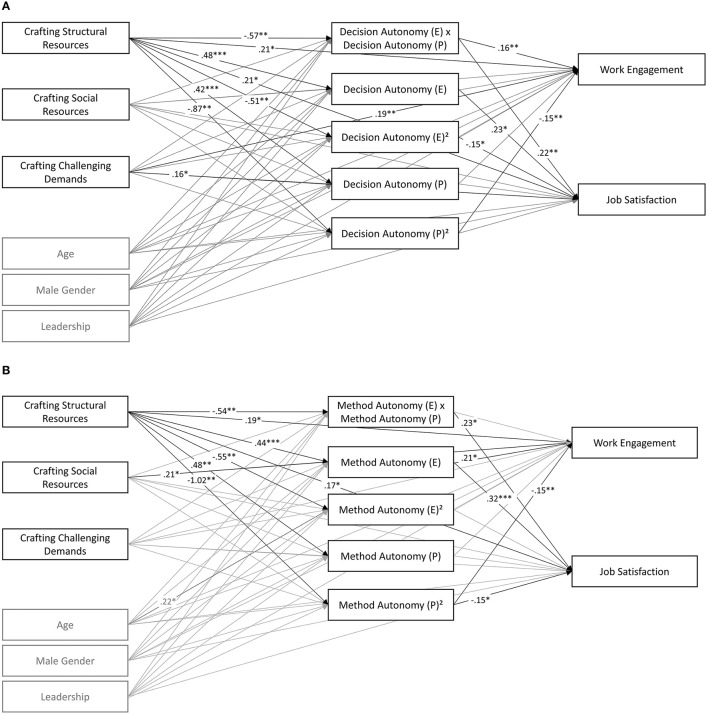
Pathmodel 1: fit between ideal decision (method) autonomy as a mediator between job crafting and work engagement/job satisfaction. **(A)** Method autonomy. **(B)** Decision autonomy. All estimated lines are displayed and colored according to their significance (gray: *p* ≥ 0.05; black: *p* < 0.05). Black dotted lines show the total effects that were significant. All models controlled for age, gender, and leadership. ^*^p < 0.05; ^**^p < 0.01; ^***^p < 0.001.

The results (Tables 4, 5, Figure 2) showed a significant indirect effect of crafting structural resources on work engagement and job satisfaction, mediated through ideal and perceived decision autonomy (_WE_ = 0.13; _JS_ = 0.14) and method autonomy (_WE_ = 0.14; _JS_ = 0.18). The indirect effects of crafting social resources or challenges were not significant. The ß-weights between independent variables and mediator variables showed a problem with high multicollinearity between the mediator variables. However, this multicollinearity must be tolerated due to the methodological requirements resulting from the aim to investigate the P-E fit. The overall fit was good, so the indirect effect could be interpreted cautiously.

**Table 4:** Results of path model predicting work engagement and job satisfaction by job crafting mediated through autonomy fit.

	**Model with decision autonomy**							**Model with method autonomy**						
	**Autonomy (E) x (P)**	**Autonomy (E)**	**Autonomy (P)**	**Autonomy (E)** ^2^	**Autonomy (P)** ^2^	**Work engagement**	**Job satisfaction**	**Autonomy (E) x (P)**	**Autonomy (E)**	**Autonomy (P)**	**Autonomy (E)** ^2^	**Autonomy (P)** ^2^	**Work engagement**	**Job satisfaction**
Structural resources crafting	−0.57^**^	0.48^***^	0.42^***^	−0.51^**^	−0.87^**^	0.21^*^	0.21^*^	−0.54^*^	0.44^***^	0.48^***^	−0.55^**^	−1.02^**^	0.19^*^	0.17^*^
Challenging demands crafting	0.03	−0.01	0.16^*^	0.11	−0.04	0.19^**^	0.04	0.01	0.04	0.13	0.12	−0.04	0.18^*^	0.02
Social resources crafting	0.04	−0.04	−0.05	0.00	0.02	0.01	0.04	0.01	0.02	−0.04	0.02	0.00	0.00	0.03
Age	0.11	−0.05	0.00	0.13	0.11	0.08	0.03	0.01	−0.06	0.01	0.22^*^	0.08	0.08	0.03
Male gender	0.03	0.06	0.02	−0.05	0.06	0.08	−0.02	0.02	0.04	−0.02	−0.09	0.08	0.08	0.00
Leadership responsibility	0.14	0.07	0.05	0.01	0.09	−0.06	−0.08	0.07	0.01	0.01	−0.04	0.03	−0.06	−0.06
Autonomy (E) x (P)						0.16^**^	0.22^**^						0.14	0.23^*^
Autonomy (E)^2^						−0.07	−0.15^*^						0.01	−0.05
Autonomy (E)						0.13	0.23^*^						0.21^*^	0.32^***^
Autonomy (P)^2^						−0.15^**^	−0.10						−0.15^**^	−0.15^*^
Autonomy (P)						−0.02	−0.02						−0.05	−0.03
a1						0.12	0.21^**^						0.16	0.27^*^
a2						−0.05	−0.03						0.00	0.03
a3						0.15	0.25^***^						0.26	0.38^**^
a4						−0.38^***^	−0.47^***^						−0.29^***^	−0.43^***^

All results are controlled for gender, age, and leadership role. N = 262. ^*^p < 0.05; ^**^p < 0.01; ^***^p < 0.001.

**Table 5:** Mediational paths.

	**Autonomy**	
**Total indirect effects**	**Decision**	**Method**
Structural resources crafting → Work engagement	0.13^**^	0.14^**^
Challenging demands crafting → Work engagement	0.00	0.01
Social resources crafting → Work engagement	0.00	0.01
Structural resources crafting → Job satisfaction	0.14^**^	0.18^***^
Challenging demands crafting → Job satisfaction	−0.01	0.01
Social resources crafting → Job satisfaction	0.00	0.01

^**^p < 0.01; ^***^p < 0.001.

In order to test the increasing effect of job crafting behavior on the fit between ideal and perceived decision (method) autonomy, Bednall and Zhang's (2020) approach to predicting the directional difference was used. Accordingly, the effect of job crafting on autonomy fit was assessed by testing the significance of the regression weight _diff_ = _E_ – _P_, where _P_ was the regression weight of job crafting as a predictor of ideal autonomy, and _E_ the weight as a predictor of perceived autonomy. Using the delta method, implemented in the lavaan package (Rosseel, 2012), the analyses showed a significant effect of crafting challenging resources on the directional difference between perceived and ideal decision autonomy (ß = −0.17, *p* < 0.05) but not regarding method autonomy. The regression weight of ideal decision autonomy (ß = 0.16, *p* < 0.05) was significant and positive, but the weight of perceived decision autonomy (ß = −0.01, n.s.) was insignificant, which contradicted the proposed theory. Crafting structural and social resources showed no significant effects (Table 6).

**Table 6:** Effects of job crafting behaviors on the fit between perceived and ideal autonomy.

	**Person-environment fit**	
	**Decision autonomy**	**Method autonomy**
	***ß***	***ß***
Structural resources crafting	0.06	−0.05
Challenging demands crafting	−0.17^*^	−0.09
Social resources crafting	0.01	0.06

ß = ß_E_ – ß_P_. ^*^p < 0.05.

**Figure 3:**
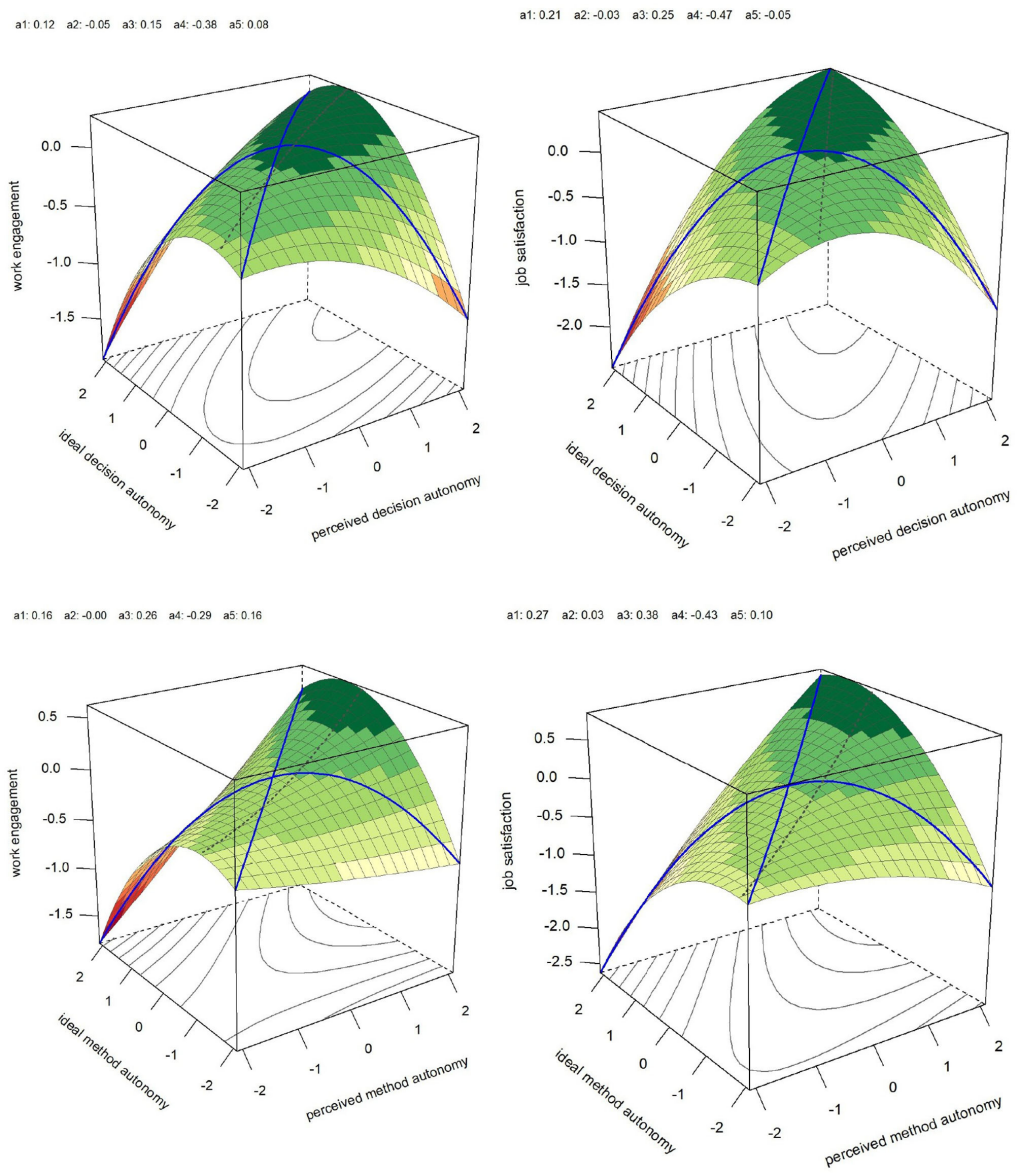
Response surface of the perceived level of autonomy (E) and the Person's ideal level (P) predicting work engagement and job satisfaction. All models controlled for age, gender, and leadership. Blue lines display lines of congruence and incongruence.

Additionally, the effects of fit on work engagement and job satisfaction were tested. The surface parameters were calculated and tested for significance using the approach of Shanock et al. (2010) (Table 4 and Figure 3 for results). To assess the impact of (mis-)fit, the slope and curvature along the line of congruence and incongruence were relevant. Regarding decision autonomy, the curvature along the line of incongruence was significant for work engagement (a_4_ = −0.38, *p* < 0.001) and job satisfaction (a_4_ = −0.47, *p* < 0.001). Additionally, the parameter a_1_ indicating the slope at the line of congruence was significant for job satisfaction (a1 = 0.21, *p* < 0.01) but not for work engagement, showing the positive association of the fit at a higher decision autonomy level with higher job satisfaction. Regarding method autonomy, the curvature along the line of incongruence was significant for work engagement (a_4_ = −0.29, *p* < 0.001) and job satisfaction (a_4_ = −0.43, *p* < 0.001). Additionally, the parameter a_1_ indicating the slope at the line of congruence was significant for job satisfaction (a_1_ = 0.29) but not for work engagement, showing the positive association of the fit at a higher decision autonomy level with higher job satisfaction.

Consequently, H2 stating that job crafting behavior predicts higher work engagement and job satisfaction mediated by better decision and method autonomy fit was not confirmed. However, the positive relations between P-E fit and work engagement/job satisfaction were confirmed.

### 4.4. Hypothesis 3

H3, predicting higher criticality of job crafting behavior, was tested by comparing the group means of the different types of job crafting behaviors between SMOs and non-SMOs. Controlling for age, gender, leadership, organizational size, and tenure, crafting social resources [*F*_(1, 223)_ = 10.43, *p* < 0.01, *d* = 0.45], seeking challenging demands [*F*_(1, 206)_ = 6.47, *p* < 0.05, *d* = 0.35], and crafting structural resources [*F*_(1, 206)_ = 13.59, *p* < 0.001, *d* = 0.51] were more prevalent in SMOs (Table 7 for group means).

**Table 7:** Group differences between SMOs and Non-SMOs.

**Variable**	**M (SD) SMO**	**M (SD) non-SMO**	***F* value**	***p*-value**	**Cohen's d**
Structural resources crafting	4.25 (0.47)	3.90 (0.68)	13.59	<0.001	0.51
Social resources crafting	3.51 (0.78)	3.18 (0.84)	10.43	0.001	0.45
Challenging demands crafting	3.70 (0.74)	3.35 (0.85)	6.48	0.012	0.35
Error learning	4.21 (0.65)	3.82 (0.72)	13.09	<0.001	0.50
Error risking	4.00 (0.77)	3.38 (0.83)	23.75	<0.001	0.67
Error strain	2.58 (0.83)	3.03 (0.8)	13.14	<0.001	0.50
Work engagement	5.41 (0.94)	5.04 (1.13)	7.11	0.013	0.37
Job satisfaction	5.72 (0.98)	5.01 (1.41)	14.87	<0.001	0.53

n_SMO_ = 78, n_non − SMO_ = 167.

Additionally, the relationships between job crafting behaviors and work engagement (job satisfaction) were compared between the SMO and non-SMO groups, as these variables were critical in SMOs. Firstly, work engagement and job satisfaction were regressed on the job crafting behaviors and their interactions with the group variable of SMO vs. non-SMO, controlling for age, gender, and leadership. The interaction effect of crafting social resources and the group variable on work engagement was marginally significant, indicating a stronger relationship in SMOs (Table 7). The other interactions were not significant. Secondly, the model was estimated separately for both groups (Table 8) to analyze the significant interaction effect further. The group difference of the regression weight of crafting social resources was significant (_SMO_ = 0.18, _nonSMO_= −0.06, z-value_Δ_ = 1.80, *p* < 0.05)^2^, pointing in the assumed direction. The differences in the regression weights of crafting structural resources and challenging demands were not significant. Interestingly, crafting structural resources and increasing challenging demands were significant predictors of work engagement in SMOs, whereas only increasing structural resources was significant in non-SMOs. Regarding job satisfaction, there were no significant interaction effects. The group comparison showed no significant group differences regarding the ß-weights of the three job crafting dimensions. Consequently, H3 could not be confirmed: Although the levels of job crafting were higher in SMOs, there were only hypothesis-confirm group differences in the relationships between crafting social resources and challenging demands and work engagement, but not regarding job satisfaction.

**Table 8:** Relations of job crafting compared between SMOs and non-SMOs.

		**WE**			**JS**		
		**all**	**SMO**	**non-SMO**	**all**	**SMO**	**non-SMO**
Model job crafting	Structural resources crafting	0.36^***^	0.33^*^	0.38^***^	0.33^***^	0.27	0.36^***^
	Social resources crafting	0.01	0.18	−0.06	0.02	0.08	−0.01
	Challenging demands crafting	0.20^*^	0.31^*^	0.14	0.00	0.09	−0.05
	SMO x structural resources crafting	−0.04			−0.04		
	SMO x challenging demands crafting	0.08			0.08		
	SMO x social resources crafting	0.10.			0.04		
	SMO	0.03			0.18^**^		
Model error orientation	Error learning	0.13.	0.02	0.18.	0.13.	0.09	0.16
	Error risking	0.16^*^	0.36^**^	0.06	0.10	0.22^*^	0.03
	Error strain	−0.16^*^	0.02	−0.25^**^	−0.11	0.12	−0.21^*^
	SMO x error learning	−0.07			−0.05		
	SMO x error risking	0.14.			0.12.		
	SMO x error strain	0.12.			0.13^*^		
	SMO	0.05			0.18^**^		

N_all_ = 230, n_SMO_ = 73, n_non − SMO_ = 157. All results were controlled for gender, age, and leadership role. .*p* < 0.10; ^*^p < 0.05; ^**^p < 0.01; ^***^p < 0.001.

### 4.5. Hypothesis 4 and 5

**Figure 4:**
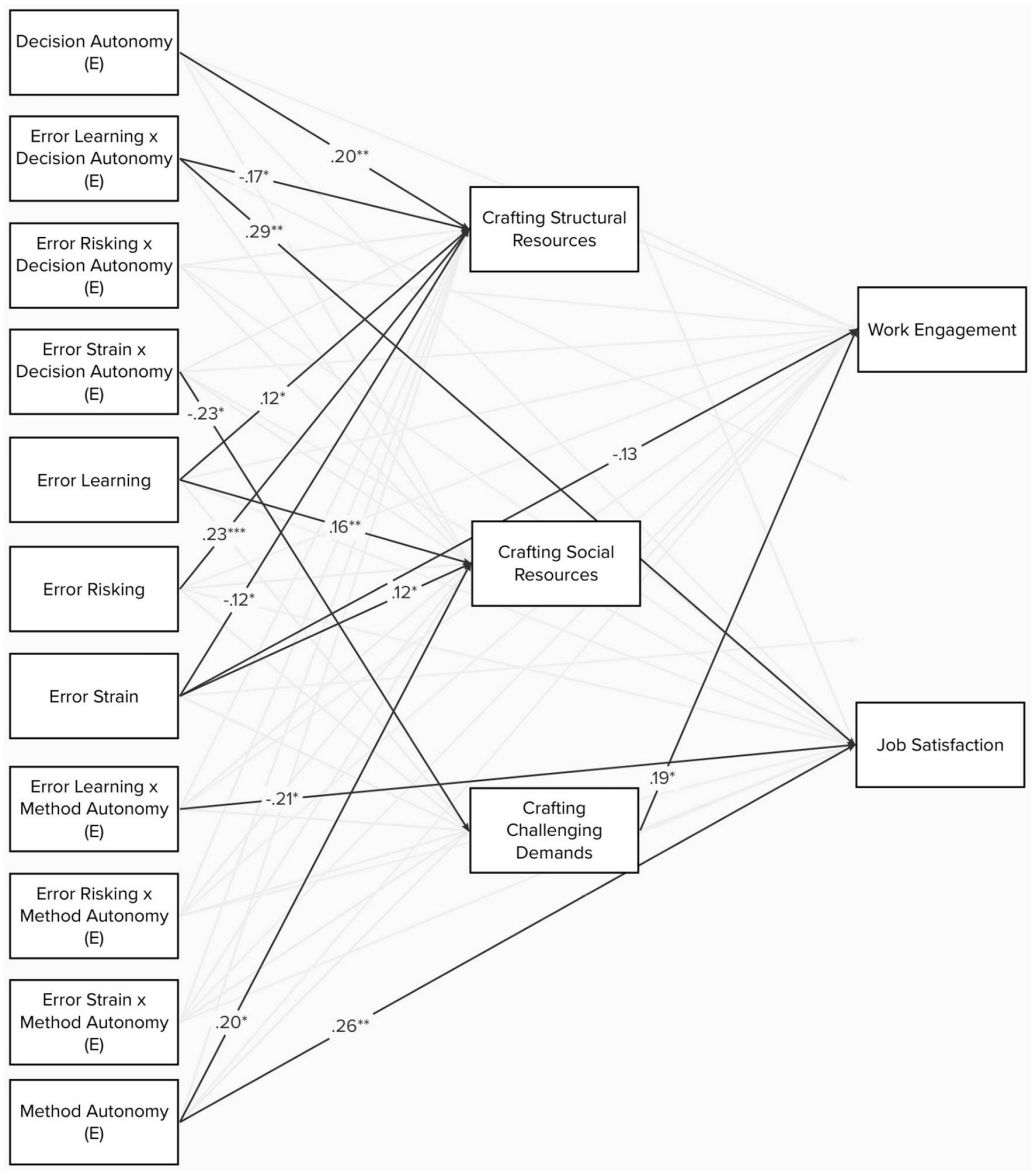
Pathmodel 2: the moderating effect of error orientation on the effect of decision and method autonomy on job crafting behaviors. All estimated lines are displayed and colored according to their significance (gray: *p* ≥ 0.05; black: *p* < 0.05). Black dotted lines show the total effects that were significant. The model was controlled for age, gender, and leadership; the covariates were included like in the smaller models (Figure 5), but not displayed due to clarity. .*p* < 0.10; ^*^p < 0.05; ^**^p < 0.01; ^***^p < 0.001.

In order to examine H4 and H5, path analysis based on the lavaan package in R (Rosseel, 2012) was used. Robust estimators were used as multivariate normal distribution was violated. Each type of job crafting was predicted by method and decision autonomy, the three dimensions of error orientation, and their corresponding interactions. Job crafting, in turn, predicted work engagement and job satisfaction. Direct effects of the predictors on the outcomes were allowed, and the model was controlled for age, gender, and leadership role. Fit indices were not interpretable as the model was just identified (*df* = 0). The results are shown in Table 9, and Figure 4 presents the path model. The models showed two significant interaction effects: error strain interacted with decision autonomy in its effect on crafting challenging demands (ß = −0.23, *p* < 0.05), and learning from errors showed a significant interaction effect with decision autonomy on crafting structural resources (ß = −0.17, *p* < 0.05). Additionally, learning from errors showed a significant main effect on crafting social resources (ß = 0.16, *p* < 0.01). Risking errors was a significant predictor of crafting structural resources (ß = 0.23, *p* < 0.001) and challenges (ß = 0.31, *p* < 0.001). Error strain was a negative predictor of crafting structural resources (ß = −0.12, *p* < 0.05) but a positive one of crafting social resources (ß = 0.12, *p* < 0.05). There were no significant interactions of error orientation dimensions and method autonomy regarding the prediction of job crafting behaviors.

**Table 9:** Work engagement, job satisfaction, and job crafting behavior predicted by perceived decision and method autonomy.

	**Structural resources crafting**	**Social resources crafting**	**Challenging demands crafting**	**Work engagement**	**Job satisfaction**
Structural resources crafting				0.17.	0.17.
Social resources crafting				0.02	0.03
Challenging demands crafting				0.19^*^	0.00
Decision autonomy (E)	0.20^**^	−0.09	0.07	0.06	0.09
Error learning x decision autonomy (E)	−0.17^*^	0.10	0.07	0.09	0.29^**^
Error risking x decision autonomy (E)	−0.11	−0.06	−0.24	−0.04	−0.15
Error strain x decision autonomy (E)	−0.12	−0.08	−0.23^*^	0.04	0.08
Error learning	0.12^*^	0.16^**^	0.04	0.02	0.03
Error risking	0.23^***^	−0.01	0.31^***^	0.01	0.06
Error strain	−0.12^*^	0.12^*^	−0.07	−0.13^*^	−0.07
Error learning x method autonomy (E)	0.06	−0.12	−0.04	−0.17	−0.21^*^
Error risking x method autonomy (E)	0.06	−0.02	0.17	0.03	0.04
Error strain x method autonomy (E)	0.04	0.01	0.02	0.02	0.00
Method autonomy (E)	0.14.	0.20^*^	0.15	0.14	0.26^**^
***Total effects***				**Work engagement**	**Job satisfaction**
Decision autonomy (E)				0.11	0.12
Error learning x decision autonomy (E)				0.08	0.27^*^
Error risking x decision autonomy (E)				−0.11	−0.17
Error strain x decision autonomy (E)				−0.03	0.06
Error learning				0.05	0.05
Error risking				0.10	0.10
Error strain				−0.16^**^	−0.09
Error learning x method autonomy (E)				−0.17.	−0.21^*^
Error risking x method autonomy (E)				0.07	0.05
Error strain x method autonomy (E)				0.03	0.01
Method autonomy (E)				0.20.	0.29^**^

All results are controlled for gender, age, and leadership role. .*p* < 0.10; ^*^p < 0.05; ^**^p < 0.01; ^***^p < 0.001.

**Figure 5:**
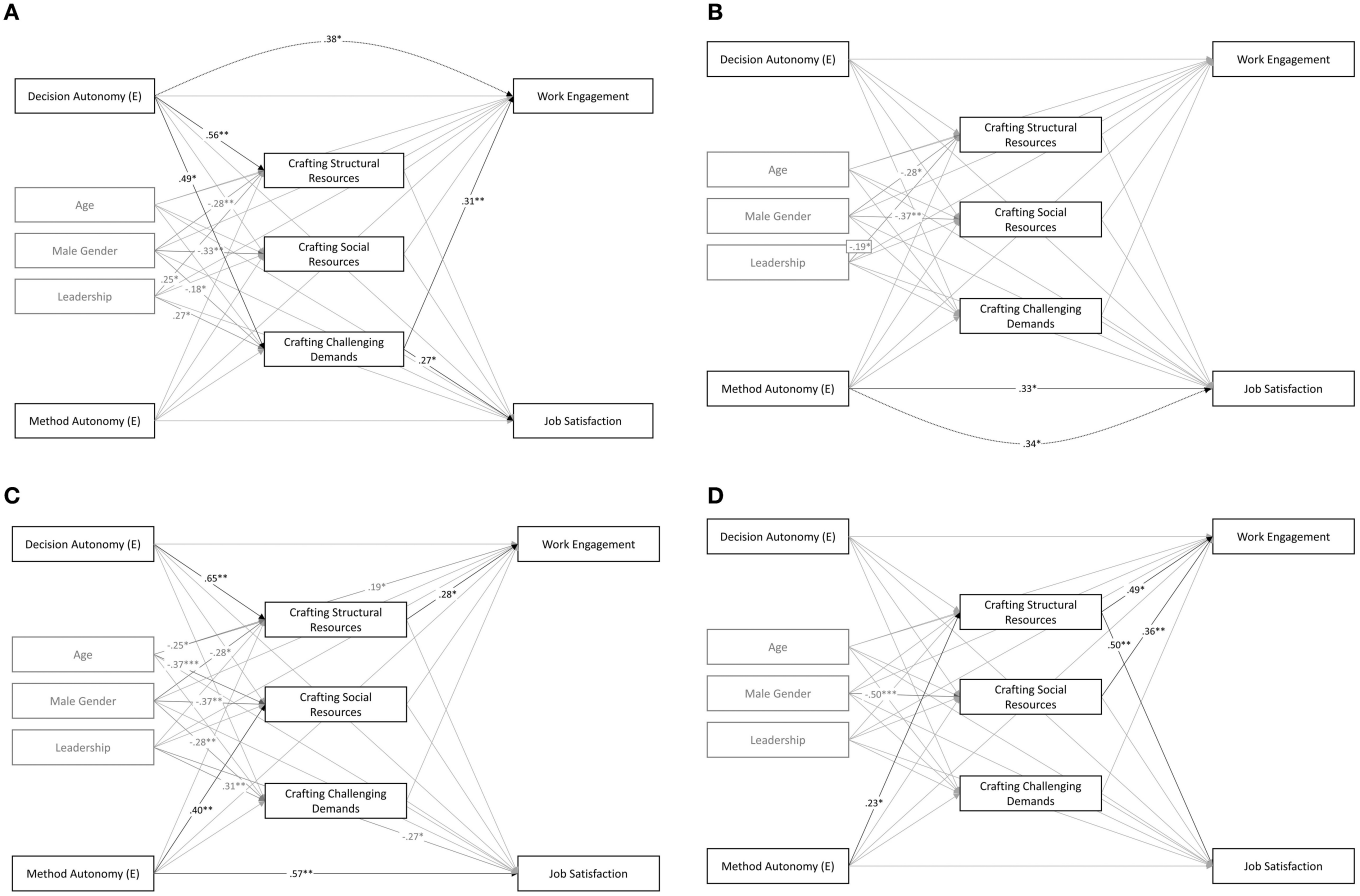
The relation between autonomy, job crafting and work engagement and satisfaction according to differences in learning from errors and strain from errors. **(A)** Low strain from error. **(B)** High strain from error. **(C)** Low learning from errors. **(D)** High learning from errors. All estimated lines are displayed and colored according to their significance (gray: *p* ≥ 0.05; black: *p* < 0.05). Black dotted lines show the total effects that were significant. All models controlled for age, gender, and leadership. .*p* < 0.10; ^*^p < 0.05; ^**^p < 0.01; ^***^p < 0.001.

In order to further examine the moderation effects, separate models based on the data of participants with high (>2nd tercile) vs. low (<1st tercile) error strain and learning from errors (Table 10) were estimated. The models were reduced to the minimum of necessary variables in order to ensure sufficient power for the smaller sample sizes of the respective high- and low-expression subgroups (*n* = 92).

**Table 10:** Work engagement, job satisfaction, and job crafting behavior predicted by perceived decision and method autonomy compared between the groups of high vs. low error learning and error strain.

		**Crafting structural resources**		**Crafting social resources**		**Crafting challenging demands**		**Work engagement**		**Job satisfaction**	
		**Low**	**High**	**Low**	**High**	**Low**	**High**	**Low**	**High**	**Low**	**High**
Error learning^a^	Crafting structural resources							0.28^*^	0.49^*^	0.02	0.50^**^
	Crafting social resources							0.11	0.09	0.00	0.13
	Crafting challenging demands							−0.09	0.36^**^	0.01	0.15
	Decision autonomy (E)	0.65^**^	0.13	−0.18	−0.02	0.05	0.12	0.08	0.25	−0.09	0.28
	Method autonomy (E)	0.03	0.23^*^	0.40^*^	0.09	0.29	0.37	0.30	−0.29.	0.57^**^	−0.19
Error strain^b^	Crafting structural resources							0.24	0.16	−0.01	0.05
	Crafting social resources							−0.03	−0.03	−0.05	0.08
	Crafting challenging demands							0.31^**^	0.03	0.27^*^	0.00
	Decision autonomy (E)	0.56^**^	0.24	0.09	−0.06	0.49^*^	−0.04	0.09	0.17	0.07	0.18
	Method autonomy (E)	0.07	0.16	0.13	0.19	0.07	0.10	−0.01	0.20	0.11	0.33^*^

All results are controlled for gender, age, and leadership role.

^a^subsample of low error learning n = 77; Subsample of high error learning n = 78.

^b^both subsamples n = 92. .*p* < 0.10; ^*^p < 0.05; ^**^p < 0.01; ^***^p < 0.001.

The results showed that in the case of low strain from error, decision autonomy was related to moderately higher crafting of challenging demands (_low_ = 0.49, *p* < 0.05), while in the case of high error strain, decision autonomy was unrelated to crafting of challenging demands (_high_ = −0.04, *p* = 0.814). The difference was significant (*z*-value_Δ_ = 1.89, *p* < 0.05). Additionally, low error strain was associated with a more positive relation between decision autonomy and crafting structural resources than high error strain was (_low_ = 0.56, *p* < 0.01 vs. _high_ = 0.24, *p* = 0.116), but the difference was only marginally significant (*z*-value_Δ_ = 1.38, *p* = 0.084). There was no group difference in the association between decision autonomy and crafting social resources; both were non-significant. The associations of method autonomy with the three job crafting dimensions did not vary significantly between the groups.

The results of the group comparison of low vs. high learning from errors showed that in the case of low learning from errors, perceived decision autonomy predicted higher crafting of structural resources (ß = 0.65, *p* < 0.01) than in the case of high learning from errors (ß = 0.13, *p* = 0.207, z-value_Δ_ = 2.19, *p* < 0.05), which contradicted H5a. In turn, the group differences in the relationships of decision autonomy with crafting social resources and challenging demands were in the expected direction but of marginal size and non-significant. In line with H5a, method autonomy was positively related to crafting structural resources in the group with high error learning (ß = 0.23, *p* < 0.05), whereas it was unrelated in the group with low error learning (ß =0.03, *p* =0.866), but the group difference was non-significant (*z*-value_Δ_ = −0.83, n.s.). In contrast to H5a, method autonomy predicted higher crafting of social resources in the group of low learning from errors (ß = 0.40, *p* < 0.05), compared to high learning from errors (ß = 0.09, *p* = 0.523). In turn, there was no group difference regarding the relation between method autonomy and crafting challenging demands. Consequently, H5 was partially confirmed but partially also disconfirmed.

In order to examine H4, proposing learning from errors (a), taking error risks (b), and low error strain (c) as moderators of the relationship between decision and method autonomy and work engagement and job satisfaction, the total effects of the path model 2 (Figure 4) were interpreted. Regarding work engagement, the interaction of method autonomy with learning from errors showed a marginal significant total effect (ß = −0.17, *p* = 0.094). The other interaction effects did not reach the significance level (Table 9). Although the comparison of the groups with low and high learning from errors showed a group difference in the ß-weights of method autonomy as a predictor of work engagement, the difference was non-significant.

The findings regarding job satisfaction were mixed: the interaction of learning from errors with decision autonomy showed a significant positive effect (ß = 0.27, *p* < 0.05), while the interaction with method autonomy was significant and negative (ß = −0.21, *p* < 0.05). The group analysis revealed a positive total effect of decision autonomy on job satisfaction in the group of high learning from mistakes, while the effect was neglectable in the low-learning-from-mistakes group (_low_ = −0.08, *p* = 0.728 vs. _high_ = 0.36, *p* = 0.055). The group difference was marginally significant (*z*-value_Δ_ = −1.47, *p* = 0.708). In turn, method autonomy showed a positive total effect on job satisfaction in the group with low learning from mistakes, whereas in the other group, the total effect was non-existent (_low_ = 0.57, *p* < 0.01, vs. _high_ = 0.00, n.s., *z*-value_Δ_ = 2.12, *p* < 0.05).

### 4.6. Mediational analyses

The explorative analyses of the mediational paths showed that the positive effect of risking errors (ß = 0.10, *p* < 0.01) as well as the negligibly small interaction effect of error strain and decision autonomy (ß = −0.07, *p* < 0.05) on work engagement were partly mediated through job crafting behaviors. The group comparison showed that in the group with low strain from errors, the effect of decision autonomy on work engagement was partially mediated by job crafting (ß = 0.28, *p* < 0.05), while in the group of high strain, no mediation was found (ß = 0.04, *p* = 0.469, z-value_Δ_ = 1.95, *p* < 0.05).

Additionally, the group comparison of high vs. low error learning yielded an interesting pattern: for employees with low learning from errors, decision autonomy was stronger related to crafting structural resources (_low_ = 0.65, *p* < 0.01 vs. _high_ = 0.13, *p* = 0.647; *z*-value_Δ_ = 2.19, *p* < 0.05), but crafting structural resources itself was related with job satisfaction only in the group of high learning from errors, but not in the group of low learning from errors (_high_ = 50, *p* < 0.01, vs. _low_ = 0.02, n.s.; *z*-value_Δ_ = −2.26, *p* < 0.05).

### 4.7. Hypothesis 6

To test H6, predicting higher criticality of the dimensions of error orientation in SMOs, firstly, the group means of the three dimensions of error orientation were compared between SMOs and non-SMOs. Controlling for age, gender, leadership, organizational size, and tenure, risking errors [*F*_(1, 206)_ = 23.75, *p* < 0.001, *d* = 0.67], learning from errors [*F*_(1, 206)_ = 19.09, *p* < 0.001, *d* = 0.50] were higher, while strain from errors [*F*_(1, 206)_ = 13.14, *p* < 0.001, *d* = 0.50] was lower in SMOs (see Table 7).

Secondly, the relevance of the three dimensions of error orientation for work engagement and job satisfaction was compared between SMOs and non-SMOs. Firstly, the full model with interaction terms of the group variable with the dimensions of error orientation, controlling for age, gender, and leadership, was estimated. Secondly, two separate models based on the subsamples of SMOs and non-SMOs were estimated (Table 8).

The model based on the full sample showed (marginal) significant interactions, making the group comparison reasonable. The group analysis showed no differences in the relationships between learning from errors with work engagement/job satisfaction between SMOs and non-SMOs. In turn, risking errors was a significant positive predictor of work engagement (_SMO_ = 0.36, *p* < 0.01; _non − SMO_ = 0.06, *p* = 0.543) and job satisfaction (_SMO_ = 0.22, *p* < 0.05; _non − SMO_ = 0.03, *p* = 0.807) among SMO employees but not among non-SMO employees. In contrast to our predictions, only in non-SMOs, strain from errors was related to less work engagement (_non − SMO_ = −0.25, *p* < 0.01; _SMO_ = 0.06, *p* = 0.881) and job satisfaction (_non − SMO_ = −0.21, *p* < 0.05; _SMO_ = 0.03, *p* = 0.189). Consequently, the results partially aligned with the hypotheses (H6b) but partially contradicted the hypotheses (H6a and c).

### 4.8. Explorative analyses

In order to understand the effect of error orientation better, the relationship between error orientation and ideal autonomy was examined. Regressing ideal decision and method autonomy on learning from errors, risking errors, and error strain under the control of age, gender, and leadership showed a significant positive relation between risking errors and ideal decision (ß = 0.19, *p* < 0.05) and method autonomy (ß = 0.21, *p* < 0.05), while learning from errors predicted higher ideal method autonomy (ß = 0.19, *p* < 0.05), but no decision autonomy (ß = 0.15, n.s.). Strain from error showed a negative, albeit non-significant, effect on both types of autonomy (_method_ = −0.08, n.s.; _decision_ = −0.12, n.s.).

## 5. Discussion

This work provides several valuable insights into the novel organizational form of SMOs and its attributes at the individual level. The current results confirm that the organizational changes in SMOs are associated with a higher perception of decision and method autonomy at the individual level (H1). Thus, the results improve the first evidence of Doblinger and Class (2023) on decision autonomy by including method autonomy and controlling for the influence of organizational age and size to prevent systematic bias. Both forms of autonomy were outstandingly high, requiring further attention when looking at the mechanisms within SMOs. However, despite the higher absolute autonomy levels in SMOs, the shares of autonomy shortage and autonomy surplus were higher in non-SMOs. This finding shows that ideal autonomy varies between individuals and that employees with high ideal decision autonomy may feel attracted to SMOs in particular, which contributes to the success of SMOs (Maier, 2013; Reitzig, 2022; Schell and Bischof, 2022) and is an important insight for the theory about SMOs.

The results did not confirm that job crafting behaviors were related to higher work engagement and job satisfaction through increased fit between ideal and perceived decision or method autonomy (H2). Nonetheless, the results gave several essential insights. Although there was a significant indirect effect of crafting structural resources on work engagement, mediated through perceived and ideal decision and method autonomy and their interactions, crafting structural resources had no significant effect on the directional difference, contradicting the assumption of an enhancing effect on the fit between ideal and perceived autonomy. Crafting structural resources was related to higher perceived and ideal decision and method autonomy, thus weakening the relation to the directional difference. This contrasts the previous findings of Tims et al. (2016), Chen et al. (2014), and Kooij et al. (2017), who found that different types of job crafting were predictive of needs-supplies and person-job fit. One possible explanation of the different findings may be using the molecular measure of fit based on the scale of Cable and DeRue (2002).

The finding that crafting challenging demands was only associated with higher ideal decision autonomy, not perceived autonomy, contradicts the hypothesized effect direction and could be explained by a reversed relationship, such as proposed by Tims and Bakker (2010): High ideal decision autonomy may cause crafting challenging demands to increase the perceived autonomy and, thus, P-E-fit. Additionally, job crafting could be a moderator, buffering the negative effect of decision autonomy (mis-)fit (Vogel et al., 2016).

The hypothesis-confirm curvilinear relationships between autonomy (mis-)fit and work engagement/job satisfaction add to the prior research of Stiglbauer and Kovacs (2018), who found already effects of decision and method autonomy (mis-)fit on flourishing and wellbeing by showing that the relationship also holds for work engagement and job satisfaction. The current findings emphasize the relevance of means to increase the P-E fit regarding decision and method autonomy, particularly in organizational transformations that increase individual job autonomy. However, according to the current results, job crafting is not necessarily the suitable method to improve P-E fit, although testing the relation in a longitudinal design and an additional measure of fit could thoroughly verify or falsify the hypothesis. The non-significant direct effect of crafting social resources on work engagement is surprising and contradicts prior findings of Moreira et al. (2022). One potential explanation for this difference might lie in the minor modification of the scale in the current study: Crafting social resources included getting advice from peers, not only from supervisors (as in the original job crafting scale).

The hypothesis of higher criticality of job crafting behaviors in SMOs (H3) was supported by the higher expression of job crafting behaviors in the SMO employees' group than in the non-SMO employees' group. While crafting social resources and crafting challenging demands showed more substantial relationships with work engagement in SMOs than in other organizations, crafting structural resources was equally related to work engagement in SMOs and non-SMOs. Hence, the latter association seems to be independent of the organizational context, which aligns with the findings on the general positive relation (Bakker et al., 2012). The insignificant group difference concerning the relationship between job crafting and job satisfaction points to other SMO-inherent factors that foster job satisfaction: for example, an excellent person-environment fit regarding autonomy caused by the selective attraction of those employees who strive for high decision autonomy (Schneider et al., 1995; Barrick and Parks-Leduc, 2019). Consequently, although job crafting is more present in SMOs, there is no evidence that it is more critical for job satisfaction but partial evidence that it may be more critical for work engagement. Nonetheless, as job crafting is more present in SMOs, it may also be more critical for other outcomes, such as burnout (Tims et al., 2013).

In contrast to the predictions of H4, method autonomy only related positively to job satisfaction in the low learning from errors group but did not relate to it in the other group. However, in line with the predictions, decision autonomy was stronger related to job satisfaction and work engagement in the case of high learning from errors compared to low learning from errors, but these effects did not reach (marginal) significance. A relieving effect of method autonomy may explain these contradictory findings, which aligns with the proposed buffering effect of job resources (Bakker and Demerouti, 2007; Xanthopoulou et al., 2007). Method autonomy may reduce the fear of making mistakes, which likely is higher when mistakes are not considered an opportunity to learn, and thus, increase satisfaction.

In contrast to the predictions, there was no evidence of a moderating effect of error strain and risking errors regarding the relationship between job autonomy and work engagement or satisfaction. Hence only learning from errors seems to influence the relationship between autonomy and work engagement.

However, the current results showed that strain from errors, learning from errors, and risking errors are associated with job crafting behaviors. The moderating effect of strain from errors was partially confirmed (H5c): for employees with low strain from error, higher decision autonomy was associated with higher crafting of structural resources and crafting of challenging demands, while for those with high strain from error, it was associated less or not at all. That aligns with the proposed theory: crafting challenges increases the possibility of mistakes, and thus high strain from mistakes may prevent employees from proactively using their decision autonomy to look for new challenges (Wei and Hisrich, 2016). In contrast to the hypothesis, decision autonomy seems to be of minor importance to crafting social resources; thus, the extent of error strain was also irrelevant. No error-strain-related group differences regarding the relationship between method autonomy and job crafting behaviors were found, again contradicting H5c. Thus, error strain may be a more serious threat when using decision autonomy compared to method autonomy.

The hypotheses about the moderating effects of learning from errors (H5a) and risking errors (H5b) regarding job crafting behaviors were not confirmed. While there was no evidence of any interaction between risking errors and decision or method autonomy (H5b), the results revealed a hypothesis-contradicting interaction effect regarding learning from errors (H5a). In the case of low learning from errors, there was a strong relation between decision autonomy and crafting structural resources, but a neglectable one when learning from errors was high. Interestingly, the results also showed that crafting structural resources was less related to work engagement in the low-error-learning group than in the high-error-learning group. Additionally, only in the case of low error learning the relationship between decision autonomy and work engagement was partially mediated through crafting structural resources. One explanation could be that employees who see errors as a learning opportunity dare to craft their job independently of receiving explicit autonomy. Employees who see errors less as learning opportunities may need decision autonomy to dare to craft structural resources, which aligns with the previous finding that a learning-oriented organizational climate was directly related to individual proactive behaviors (Caniëls and Baaten, 2019). There were no significant relations of decision autonomy with the other job crafting dimensions. However, the relations pointed in the hypothesized directions with negative relations of decision autonomy with crafting social resources in the case of low learning from errors but independence in the case of high learning from errors. A subsample of too small size may be one reason for the non-significance. Nonetheless, the effect direction is interesting: it points to the fact that if learning from errors was low, decision autonomy could be used to avoid confrontations through peer feedback. This hypothesis aligns with Aben et al.'s (2022) finding that error tolerance predicts higher feedback tolerance.

The results for H5a were also mixed regarding method autonomy: in line with H5a, for employees with high learning from errors, method autonomy was positively related to crafting structural resources, whereas both variables were unrelated in the other group. In contrast to H5a, method autonomy was positively associated with higher social resources crafting for employees with low learning from errors, whereas this relationship was neglectable for employees with high learning from errors. This finding also supports the notion that the effect mechanisms of the method and decision autonomy differ (Spiegelaere et al., 2016; Muecke et al., 2020). Muecke et al. (2020) found that feelings of responsibility mediated the relationship between decision autonomy and work engagement, whereas the relationship with method autonomy was mediated through cognitive demands. Hence, interpreting the current results, method autonomy may trigger the search for feedback by peers or supervisors to handle the cognitive demand and thus prevent mistakes when mistakes are not seen as learning opportunities. In contrast, employees with a learning attitude toward mistakes may handle the cognitive demand more easily as they can tolerate potential mistakes better. In turn, when it comes to decisions, employees with low learning from errors may fear the judgment of their peers or the exposure in the case of a potentially wrong decision in the recent past, as they feel responsible and, thus, avoid confrontation with peers (Aben et al., 2022). Additionally, instead of a moderating effect (H5c), the results identified risking errors as a predictor of increasing structural resources and challenging demands of small to moderate size, pointing to an autonomy-independent relationship between risking errors and job crafting behaviors.

In line with the predictions of H6, the comparison of the SMO employees with the non-SMO employees showed that learning from errors and risking errors was higher, and strain from errors was lower in SMOs compared to non-SMOs, pointing to a higher criticality of these attitudes in SMOs. Additionally, in line with the predictions, risking errors was related positively to work engagement and job satisfaction in SMOs but not in non-SMOs (H6b). In contrast to our predictions, strain from errors was negatively associated with work engagement and job satisfaction only in non-SMOs, but it was irrelevant in SMOs (H6c). This finding may result from a third variable associated with SMOs mitigating the relevance of strain from errors, such as psychological safety (Edmondson and Lei, 2014), a context-related variable influencing the expected consequences of making mistakes. Learning from errors showed independence of work engagement or job satisfaction in SMOs and non-SMOs (H6a). Yet learning from errors was, on average, higher in SMOs, and thus, it may be relevant for other outcomes, such as job crafting or innovative behaviors (Gu et al., 2013). Consequently, risking errors seems to be more important in SMOs than in non-SMOs, whereas the picture is not as clear regarding strain from errors and learning from errors.

### 5.1. Theoretical implications

The current work contributes in several ways to previous theories and research on job crafting, person-environment fit, error orientation, and SMOs. Firstly, the work adds quantitative evidence to the qualitative findings about the functioning of SMOs (Lee and Edmondson, 2017; Martela, 2019) by proving that method and decision autonomy are higher in SMOs, despite other inter-organizational variations. Showing that employees in SMOs differ from non-SMO employees in their behaviors and attitudes supports the controversial idea that SMOs differ significantly from other organizational forms (Martela, 2019).

The fact that these behaviors and attitudes were partially stronger related to work engagement and job satisfaction supports the notion that SMOs have different requirements for their employees. However, the exact effect is still unclear, but these insights pave the way for further investigations of the effect mechanisms.

Secondly, the current work added to the research on P-E fit by investigating potential antecedents of P-E fit, such as job crafting behavior. Based on the non-significant relationship between job crafting and work engagement, the results support the notion that the atomic measurement of P-E-fit does not equate to the molecular approach (Edwards, 2001), adding a new perspective to the previous positive findings based on a molecular approach (Cable and DeRue, 2002; Chen et al., 2014; Tims et al., 2016; Kooij et al., 2017). As previous literature argued that the atomic approach could be a more exact measurement (Edwards, 2001; Stiglbauer and Kovacs, 2018), the current results support the value of further investigating the causal relationships between job crafting and P-E fit. Additionally, the current study extended the knowledge about the relevance of P-E fit for work engagement and job satisfaction by showing that a surplus of method and decision autonomy was also associated with worse engagement and satisfaction. This supports the universality of this effect, at least when it comes to very high levels of autonomy.

Thirdly, the current work builds a first connection between the job crafting theory (Wrzesniewski and Dutton, 2001; Tims et al., 2012; Zhang and Parker, 2019) and the error orientation theory (Rybowiak et al., 1999; Keith and Frese, 2008): the results support that individual error orientation is a further relevant interindividual antecedent of job crafting that can explain interindividual variations. Importantly, the finding that it interacted with the other antecedents, decision and method autonomy, enriches the knowledge about the autonomy-job crafting relationship and can help explain potential variations in findings. The results also demonstrated that the dimensions of job crafting differ in their relationships with the dimensions of error orientation, emphasizing the need for distinction between the types of job crafting (Zhang and Parker, 2019). While learning from errors showed associations with development-related behaviors (structural resources), strain from errors was associated with crafting behaviors to reduce uncertainty (challenging demands) but increase support (social resources). Consequently, the results also deepen the understanding of the concept of error orientation (Rybowiak et al., 1999; Keith and Frese, 2008), as these different associations with job crafting and autonomy indicate that a separate consideration of the dimensions is valuable.

Lastly, the results extend the knowledge of job autonomy. The distinct relations of decision and method autonomy with dimensions of error orientation support the assumption of different types of job autonomy (Morgeson and Humphrey, 2006) and the differences in the effect mechanisms related (Spiegelaere et al., 2016; Muecke et al., 2020). It thus confirms the incremental value of a differentiating consideration of the job autonomy types.

### 5.2. Practical implications

There are several important implications for organizational practice in SMOs and potentially in other organizations with high individual job autonomy or in transition phases. Firstly, the job crafting and error orientation levels were higher in SMOs, pointing to a higher benefit in these organizations. Measures for organizational development, therefore, should add to an environment where risking errors is welcome and learning from errors is expected. Thus, employees can learn and embrace taking calculated risks. These implications align with prior research emphasizing the importance of handling mistakes constructively in SMOs but specify which aspects to focus on.

Secondly, the study showed that crafting social resources and challenging demands was related to higher work engagement in SMOs, and thus supporting employees in crafting their jobs could help engage employees in SMOs. Although it was not related to job satisfaction and thus may seem less important at first glance, it is essential as it relates to work engagement, which is particularly important in SMOs due to the need for proactive employees to work without supervisors. Thirdly, the results show the necessity of consciously addressing the high level of autonomy in SMOs. The perceived decision and method autonomy were higher in SMOs, and a misfit between ideal and perceived autonomy was related to worse work engagement and job satisfaction. Therefore, when organizations want to function as SMO, they need to address the fit of autonomy.

Fourthly, the findings regarding error orientation also provide ideas for personal development measures. Employees with low strain from errors were more likely to search for new challenges when they perceived more decision autonomy; thus, helping employees to feel less strain from errors could increase the innovativeness and pioneering of employees in SMOs, as they would look more for new challenges. The results also indicated the importance of supporting learning from errors, as otherwise increased method autonomy could lead to avoiding peer feedback, which is an essential part of SMOs (Martela, 2019; Reitzig, 2022; Schell and Bischof, 2022; Doblinger, 2023). Fifthly, the levels of work engagement and job satisfaction were higher in SMOs, showing that besides the entrepreneurial advantages related to SMOs, the organizational form was also associated with direct employee benefits, which makes SMOs more attractive to companies and their owners and managers.

### 5.3. Limitations and future research

Despite its relevant findings, this research has some limitations and scope for further research. Firstly, the measurement of P-E fit was challenging. Due to its more exact measurement, the atomic approach (measuring ideal and perceived autonomy separately) was chosen for measuring fit (Edwards, 2001). This approach created the challenge of using difference scores. The problem was resolved by including polynomials in the path analysis and testing the effect on P-E fit and the effect of P-E fit in separate steps. Latent moderated structural equation models (Cheung, 2009; Edwards, 2009; Su et al., 2019) would have been the exacter approach, but the small sample did not allow for such complex analyses. Other authors used a molecular approach (Cable and DeRue, 2002; Chen et al., 2014; Tims et al., 2016; Kooij et al., 2017), avoiding the statistical challenges, which interestingly yielded other results than the current study. However, according to Edwards (2001), that approach only shifts the responsibility of building the difference score to the participant and, thus, will not increase reliability and validity. Therefore, the approach of integrating the polynomial regression with response surface analysis into the path model was taken. Using latent moderated structural equation models based on a bigger sample size could be the scope for future research. Another issue in this context was problems with multicollinearity due to the high correlations between the autonomy variables. That reduced the related predictors' reliability, despite the affected variables' good reliability. However, the other paths could be interpreted as the model's fit was good. Although this study intended to increase validity using the atomic approach, due to the problems mentioned above, future research could also investigate the proposed relations based on a molecular approach or a longitudinal design, as this could reveal the time-delayed association of job crafting and better P-E-fit.

Secondly, as already mentioned, the study was based on a minimum sample size due to the hard accessibility of SMO employees. The sample size was sufficient for the analysis based on the whole group but was marginal for the group analysis related to the moderation hypotheses. Unfortunately, it also hindered comparing the full path model between SMO and non-SMO employees (H3, H6). Instead, less complex relations were investigated, which pointed in the right direction. Future research could test these relations in a larger sample based on the current first evidence of group-related differences. Additionally, the sociodemographic characteristics of the study samples varied to a certain degree (e.g., industries), which may have biased the comparison. However, as various industries are included in both subsamples, this potential bias is mitigated.

Thirdly, the measurement of SMO was another challenge of this study. Previous research did not provide a validated scale for measuring SMO characteristics. Therefore, we relied on a checklist (Doblinger and Class, 2023), based on the characteristics of SMOs identified by Martela (2019). This could have limited the proper selection of SMO employees as the scale was not adequately validated. However, the preselection of organizations to recruit participants from ensured the inclusion of organizations that, indeed, were SMOs. Nonetheless, a validated scale to confirm that participants belong to an SMO would further improve the significance of the results and could be the scope of future research.

Fourthly, this study relied on a cross-sectional design, which does not allow for detecting time-delayed relationships, let alone causalities, which may have explained better the relationship between job-crafting behavior and autonomy fit. Therefore, the interpretation of the results is limited as only relationships could be described. Nonetheless, the detected relationships provide first essential insights into the associations between relevant variables for the different groups and thus can trigger future incremental research.

Lastly, the current study also shows the scope for future research. A further investigation of the relationship between job crafting and P-E fit could be valuable. On the one hand, the potentially reverse relationship between P-E fit and job crafting could be investigated by testing a theoretical model where ideal decision autonomy causes crafting challenging demands to increase the perceived autonomy and, thus, reach a better P-E fit. Similarly, investigating job crafting as a moderator of the effect of (mis-)fit on work engagement and job satisfaction promises valuable insights. Additionally, as job crafting is more present in SMOs, it may also be more critical for other outcomes, such as burnout, which could be subject to future studies. Another interesting research focus could be the exploration of other levers than job crafting that may help increase P-E fit. Moreover, future research should investigate the role of learning from errors in SMOs more in depth, for instance, by relating it to innovative behavior.

### 5.4. Conclusion

The results support that P-E-fit regarding job autonomy is important for work engagement and job satisfaction and that this fit likely occurs at a higher autonomy level in SMOs. Job crafting is more prevalent in SMOs but does not necessarily relate to P-E-fit. Although the dimensions of error orientation relate to job crafting, they differ in their ways of relating: While error strain and error learning interact with the effect of decision and method autonomy, risking errors did not interact but was directly related to job crafting and even with work engagement in SMOs. Reducing error strain but increasing error learning and risking errors could help increase job crafting and work engagement in SMOs or also other organizations where individual autonomy is high.

## Data availability statement

The raw data supporting the conclusions of this article will be made available by the authors, without undue reservation.

## Ethics statement

The studies involving human participants were reviewed and approved by Ethikkommission der Fakultät für Verhaltens- und Empirische Kulturwissenschaften der Ruprecht-Karls-Universität Heidelberg. The patients/participants provided their written informed consent to participate in this study.

## Author contributions

The author confirms being the sole contributor of this work and has approved it for publication.

## Footnotes

^1^All factors make unique contributions, allowing for usage as single predictors (Farnese et al., 2020).

^2^Comparisons were made by applying the formula of proposed by Clogg et al. (1995).
